# Extra-corporeal life support for near-fatal multi-drug intoxication: a case report

**DOI:** 10.1186/1752-1947-5-231

**Published:** 2011-06-23

**Authors:** Roberto Rona, Barbara Cortinovis, Roberto Marcolin, Nicolò Patroniti, Stefano Isgrò, Chiara Marelli, Roberto Fumagalli

**Affiliations:** 1 Dipartimento di Medicina Perioperatoria e Terapia Intensiva, Azienda Ospedaliera San Gerardo di Monza, via Pergolesi 33, Milan, Italy; 2 Dipartimento di Medicina Sperimentale, Università degli Studi Milano Bicocca, Azienda Ospedaliera San Gerardo di Monza, via Pergolesi 33, Milan, Italy

## Abstract

**Introduction:**

Severe mixed β-blocker and calcium channel blocker intoxication presents a significant risk for patient mortality. Although treatment is well-established, it sporadically fails to support the patient through massive overdoses, thus requiring non-conventional treatments. We report the use of extra-corporeal life support in a patient with refractory hemodynamic impairment due to multi-drug intoxication. Although sometimes used in clinical practice, extra-corporeal membrane oxygenation for intoxications has rarely been reported.

**Case presentation:**

A 36-year-old Caucasian man presented to our hospital with refractory hypotension, severe cardiac insufficiency and multi-organ failure due to mixed intoxication with atenolol, nifedipine, Lacidipine and sertraline. Together with standard treatment, we performed extra-corporeal membrane oxygenation to overcome refractory cardiogenic shock and lead the patient to achieve a full recovery.

**Conclusion:**

Standard of care for β-blocker and calcium channel blocker intoxication is well-defined and condensed into protocols of treatment. Although aimed at clearing the noxious agents from the patient's system, standard measures may fail to provide adequate hemodynamic support to allow recovery. In selected cases, extra-corporeal membrane oxygenation could be considered a bridge to drug clearance while preventing multi-organ failure due to profound shock.

## Introduction

β-blocker (BB) and calcium channel blocker (CCB) are the most common cardiovascular medication classes reported in the database of the American Association of Poison Control Centers Toxic Exposure Surveillance System [1]. BB overdose represents about 1% of patients with drug intoxication admitted to intensive care units. Fatalities are rare but not negligible, with a reported rate of about 0.5% and about 20 deaths per year in the United Kingdom. CCB and BB poisoning represent more than 65% of overall deaths caused by cardiovascular medications [2].

The most severe forms, which account for 20% of all BB overdoses, are usually due to propranolol ingestion because of its rapid absorption. In the American experience, propranolol is responsible for the majority of fatal cases (70%), followed by atenolol (20%). The patients' clinical presentation and care in cases of significant BB overdose are a direct consequence of cardiovascular depression and the need to reverse it. Critical factors are timing, type and dosage of the product ingested, the presence of a synergistic co-ingestant, medical co-morbidities and the patient's intent in ingesting the medications.

The presence of CCB could make patients particularly vulnerable to BB toxicity, because their physiological and toxic effects are similar [2]. Co-ingestion of psychotropic agents can also worsen the patient's prognosis because of respiratory depression [3].

Treatment usually consists of specific and non-specific measure aimed at stabilizing the patient while attempting to clear the poisoning from the patient's system. However, since timing is essential to preventing multi-organ deterioration and failure due to profound refractory cardiogenic shock, a standard approach might not provide adequate hemodynamic support to achieve this goal until there is complete drug clearance from the patient's system. In this regard, extra-corporeal membrane oxygenation (ECMO) might offer a valuable bridge to drug clearance while protecting organ function from irreversible damage.

## Case presentation

A 36-year-old Caucasian man with a history of hypertension, depression, bipolar disorder and two past suicide attempts was brought to our emergency department two hours following voluntary ingestion of a total estimated amount of 10 g of atenolol in association with an unknown amount of sertraline, nifedipine, Lacidipine and fluoxetinee.

Upon arrival, he presented with a decreased level of consciousness, shortness of breath, hypoxemia (arterial oxygen saturation, 91%) and hypotension (blood pressure (BP), 80/40 mmHg; heart rate (HR), 80 beats/minute). A 12-lead electrocardiogram (ECG) showed normal sinus rhythm, PR interval 168 milliseconds, prolonged corrected QT time 448 milliseconds and QRS widening 133 milliseconds. The results of his routine laboratory tests, including glucose, an electrolyte panel, liver function tests and coagulation were normal, with the exception of mild renal impairment (blood urea nitrogen 46 mg/100 mL, creatinine 2.2 mg/100 mL).

Briefly afterward the patient's condition suddenly deteriorated, and he experienced cardiac arrest. He was resuscitated according to advanced cardiac life support (ACLS) protocols, and an effective cardiac rhythm was regained 30 minutes following the cardiac arrest. However, he had severe hemodynamic instability, persistent metabolic acidosis and shock (BP 70/40 mmHg, HR 80 beats/minute), despite adequate fluid resuscitation, the administration of 100 mEq sodium bicarbonate IV bolus, very high-dose dopamine (50 μg/kg/minute) and epinephrine (2.5 μg/kg/minute) infusion.

At the same time, we started specific medical management for BB and CCB intoxication, including gastric lavage followed by gastric administration of 40 g of activated charcoal, 40 ml IV bolus injection of calcium chloride, and 5 mg glucagon IV bolus injection performed. Two hours after admission to the emergency department, the patient was transferred to our intensive care unit (ICU).

Upon arrival to the ICU, the patient was unconscious (Glasgow Coma Scale score 3) without sedation and on controlled mechanical ventilation. He had persistent shock with multiple organ failure, refractory metabolic acidosis (pH 7.22, arterial partial pressure of oxygen (PaO_2_) 79 mmHg, arterial partial pressure of carbon dioxide 49 mmHg, base excess -7.4, lactate 9.3 mmol/L) and oligo-anuric renal failure.

The specific treatment of his drug overdose was performed as follows: (1) 40 g of activated charcoal administration through a nasogastric tube three times daily for 24 hours, (2) whole bowel irrigation with 2 L of polyethylene glycol, (3) calcium chloride continuous IV infusion to provide a stable slightly supra-normal serum ionized calcium level (2 mmol/L), (4) glucagon continuous IV infusion at a rate of 5 mg/hour for 20 hours following the first 5 mg loading dose, (5) high-dose insulin and glucose continuous IV infusions (0.7 to 1.5 IU/kg/hour and 50 to 100 g/hour, respectively) and (6) single plasma exchange treatment followed by three days of high-volume continuous veno-venous hemofiltration (HV-CVVH) at a 70 to 80 mL/kg/hour ultra-filtration rate.

His plasma levels of anti-psychotic medications and CCB were reduced by medical treatment and plasma exchange. His atenolol level continued to rise for six to eight hours after toxic ingestion and then started to decrease with the initiation of HV-CVVH. His atenolol plasma level normalized after 72 hours, at which point the ultra-filtration rate was reduced to 40 mL/kg/hour. The efficacy of atenolol removal by HV-CVVH can be assessed on the basis of the trends in plasma and ultra-filtrate levels outlined in Table 1.

**Table 1:** Drug, plasma and ultra-filtrate levels and clearance^a^

	Emergency Department arrival 60 minutes after drug ingestion	After plasma exchange therapy 8 hours after drug ingestion		After 72 hours of HV-CVVH	
	
Medication	Plasma levels	Plasma levels	Ultra-filtrate	Plasma levels	Ultra-filtrate
Sertraline, μg/mL	0.55	-	-	-	-
Nifedipine, μg/mL	2.23	0.45	-	-	-
Lacidipine, μg/mL	0.34	-	-	-	-
Fluoxetinee, μg/mL	0.02	-	-	-	-
Atenolol, μg/mL	3.95	14.8	6.5	2.1	0.6

^a^HV-CVVH, high-volume continuous veno-venous hemofiltration.

The table summarizes plasma sertraline, nifedipine, lacepidine, fluoxetinee, and atenolol levels upon Emergency Department (ED) arrival. It also shows the plasma and ultra-filtrate levels, respectively, following plasma exchange and high-volume continuous veno-venous hemofiltration

The patient's cardiac insufficiency required the insertion of a pulmonary artery catheter to guide hemodynamic optimization, increasing the infusion of inotropes and vasopressors up to extremely high-dose dopamine (300 μg/kg/minute) and subsequent epinephrine (15 μg/kg/minute) and vasopressin (0.03 IU/minute) continuous infusion, which still did not achieve satisfactory hemodynamic stability or adequate peripheral tissue perfusion (BP 80/40 mmHg, HR85 beats/minute, cardiac output (CO) 4.2 L/minute, mixed venous oxygen saturation (SvO_2_) 61%, pH 7.18, and lactate 8 mmol/L). An echocardiogram confirmed global cardiac hypokinesis and depressed left ventricular function.

The patient's young age, relative lack of pre-existing co-morbidities, potassium levels and failure to accomplish satisfactory correction of tissue acidosis led us to consider extra-corporeal cardiopulmonary support. Two hours after admission extra-corporeal cardiopulmonary support (Jostra RotaFlow Centrifugal Pump; Maquet, *Jostra *Medizintechnik AG, *Hirrlingen*, Germany) was started through percutaneous femoral artery and vein cannulation. Extra-corporeal blood flow was initially set at 4 L/minute, and gas flow through an artificial lung was initiated at 2 L/minute of oxygen.

After starting ECMO, vasopressin was discontinued and both dopamine and epinephrine administration were quickly tapered (Table 2). His clinical signs of tissue perfusion improved, minimal spontaneous diuresis resumed and lactic acidosis was corrected. After 24 hours of combined ECMO and HV-CVVH treatment, his lactate levels had decreased to near physiological levels (< 2 mmol/L). Specific β-agonist cathecolamines, such as isoproterenol and dobutamine, were added to epinephrine while dopamine was replaced with a more specific vasoconstrictor, such as norepinephrine.

**Table 2:** Time course and dosage of inotropes and vasopressors during ECMO^a^

	Time since admission, hours											
	
Medication	0 to 2	2 to 4	4 to 6	6 to 8	8 to 10	10 to 12	14 to 16	18 to 20	22 to 24	36 to 38	40 to 42	46 to 48
Dopamine, μg/mL	50	**300**	150	100	50	30	25	-	-	-	-	-
Epinephrine, μg/mL	**15**	15	15	15	3	3	1	0,4	0,5	-	-	-
Noradrenaline, μg/mL	-	-	-	-	-	0.2	0.2	0.2	0,2	**0.3**	0.05	-
Dobutamine, μg/mL	-	-	-	-	-	8	8	12	**16**	8	2	-
Isoproterenol, μg/mL	-	-	-	-	-	-	-	-	**5.5**	5.5	0.5	-
ECMO												

^a^ECMO, extra-corporeal membrane oxygenation.

Unexpectedly, the patient's severe myocardial impairment was never associated with rhythm disturbance. Serial 12-lead ECGs revealed moderate sinus bradycardia, but neither atrio-ventricular blocks nor QRS widening nor cQT interval prolongation was detected, except on the admission ECG. Therefore, there was no indication for cardiac pacing.

After 24 hours, a test of extra-corporeal blood flow reduction revealed persistent cardiac insufficiency complicated by pulmonary edema (BP 85/40 mmHg, CO 3.9 L/min, pulmonary capillary wedge pressure 21 mmHg, SvO_2 _63% and ratio of PaO_2 _to fraction of inspired O_2 _< 150).

**Figure 1:**
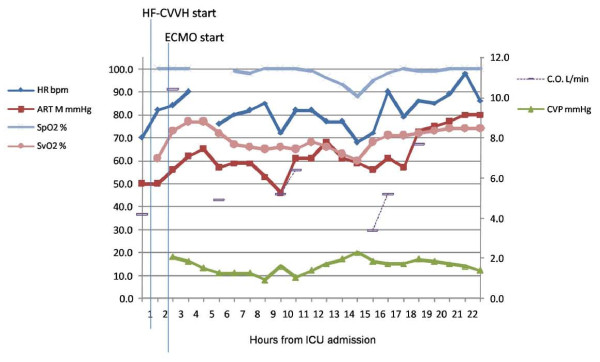
**Hemodynamic parameters prior and after extra-corporeal membrane oxygenation start for the first 24 hours**. ART M = mean arterial pressure; CO = cardiac output; CVP = central venous pressure; HR = heart rate; SpO_2 _= arterial oxygen saturation; SvO_2 _= mixed venous oxygen saturation.

The patient was weaned from ECMO 48 hours later after a successful flow reduction test. After by-pass interruption, inotropes and vasopressors were progressively tapered and discontinued completely on day 6, when his hemodynamic parameters were completely within normal range (Figure 1 and Table 2).

The second clinically relevant problem was the extremely low potassium plasma level on admission (< 0.8 mEq/L). Potassium was supplemented by high-dose potassium chloride continuous IV infusion at a rate of 30 to 35 mEq/hour without successful response. Once the by-pass was started, the effects of potential sudden cardiac death due to arrhythmia secondary to electrolyte derangement was reduced, thus the IV infusion was quickly tapered to 10 to 15 mEq/hour without cardiac rhythm disturbance. The plasma level of potassium normalized only about 12 hours later. Transient hyperkalemia was also noted with no ECG abnormalities.

We also observed progressive rhabdomyolysis (myoglobinuria, creatine phosphokinase 24,169 IU/mL and myoglobinemia 14,444 IU/mL) and some skin sub-ischemic injuries on the right gluteus and second and third left toes. The patient's rhabdomyolysis was managed with high fluid washout and to some extent by HF-CVVH. His external lesions were dressed daily. All complications resolved without sequelae.

Moderate neurological impairment progressively developed as a result of the initial anoxic insult. Basal brain CT and magnetic resonance imaging scans showed bilateral ischemic dyencephalic lesions. Serial electroencephalograms confirmed depressed cortical activity with predominant bilateral theta-delta waves.

An echocardiogram obtained three days before the patient's discharge from the ICU showed a light left ventricular hypertrophy, normokinesis and a preserved ejection fraction (58%). The patient was transferred from the ICU on day 28 without cardiac, renal or pulmonary sequelae.

One year after his discharge from the ICU the patient was not self-sufficient and therefore was living in a long-term care facility. Although his neurologic performance overall had improved over time, he still has reduced motor skills and impaired coordination, gait ataxia and mild aphasia.

## Discussion

BBs and CCBs offer similar outcomes in patients with intoxications, considering their final effect on inhibiting calcium influx into the cells. As a consequence of β-receptor activation, the G proteins responsible for converting ATP to cyclic adenosine monophosphate are blocked, thus reducing cytosolic calcium, which is essential for muscle contraction [1,2]. On the other hand, CCBs antagonize cardiac and smooth muscle cell L-type calcium channels.

In addition, BBs act as membrane stabilizers through direct inhibition of sodium influx in myocardial cells and widening of QRS because of prolongation of phase 0 of the action potential. The clinical effects of mixed intoxication consist of profound cardiovascular depression due to cardiac contractility failure, hemodynamic deterioration due to cardiogenic shock and sinus rhythm disturbances ranging from bradycardia to various degrees of atrio-ventricular blocks and asystole [4]. Of note, in our present case, we did not observe major cardiac rhythm disturbances. The near-normal heart rate (70 to 90 beats/minute) that we observed can probably be ascribed to the intense chronotropic effect of high-dose vasoactive drug therapy that counterbalanced BB- and CCB-induced bradycardia.

Although the cardiovascular system represents the target organ, neurologic impairment could present as an expression of inadequate brain perfusion or direct sedative effects, especially of more lipophilic agents such as propranolol [4]. Although non-peculiar, coma, seizures and central respiratory depression are not entirely uncommon [5].

Pre-existing conditions such as congestive heart failure [4] and newly acquired conditions such as hyperkalemia, acidosis and co-ingestants with similar physiologic effects may further enhance toxicity. Therefore, it appears essential to implement a system to establish hemodynamic stability through phases of multi-organ dysfunction until drug metabolism and removal have been achieved.

Details about specific management of BB and CCB intoxication are beyond the aim of this work, as it is an extensive review of types and technical features of ECMO. Nevertheless, it is important to emphasize that currently available guidelines and recommendations include extra-corporeal life support (ECLS), specifically ECMO, in exceptional therapies for refractory cardiac arrest and heart failure in BB, CCB and membrane-stabilizing agents (such as tricyclic anti-depressants) [6,7].

Although not reviewed here, it is also worth to mention the literature about the use of veno-venous ECLS for poisoning-related refractory hypoxemia, such as in cases of hydrocarbon, carbon monoxide and paraquat intoxication.

Veno-arterial ECLS is mainly used for cardiodepressant drugs. Single case reports exist regarding poisoning with ibuprofen, carbamazepine, tricyclic antidepressants (TCA) and fentanyl in adult patients. Near-fatal or fatal intoxication managed with ECMO has also been reported in the literature regarding massive quinidine assumption and arsenic poisoning in pediatric patients.

With regard to CCB and BB, only a few clinical cases have been reported in the literature, both as single-drug and multi-drug intoxications [8,9], and, to our knowledge, a case series of six patients [10] who presented with massive ingestion of cardiotoxic drugs, including anti-arrhythmic agents, is up to now the largest study ever published about implementing ECMO as a bridge to standard treatment.

Early recognition of indications, together with an experienced multi-disciplinary team able to implement ECMO, appears to be related throughout all published studies and reports, to achieve the best prognosis for the patient. This underlines the importance of reporting every case, regardless of whether in the end the result was successful, so that clinicians can recognize features common to different cases and implement ECMO in accordance with a more standardized protocol.

## Conclusion

ECLS already appears in toxicology-oriented ACLS guidelines and recommendations extrapolated from small case series. This includes more common devices, such as an intra-aortic balloon pump, already widely accepted in clinical practice, as well as unconventional tools, such as veno-venous and veno-arterial ECMO to support failing organs in patients through to recovery from massive intoxication. Although still sporadically reported, the use of ECMO could be increasingly implemented in selected cases and sites, provided adequate material and technical experience are available, as major determinants in the prognosis of patients who have experienced near-fatal poisoning.

## Abbreviations

ECLS: extra-corporeal life support; ECMO: extra-corporeal membrane oxygenation.

## Consent

Written informed consent was obtained from the patient for publication of this case report and any accompanying images. A copy of the written consent is available for review by the Editor-in-Chief of this journal.

## Competing interests

The authors declare that they have no competing interests.

## Authors' contributions

RR collected and interpreted the data regarding continuous renal replacement therapy (CRRT). RR and BC reviewed the literature and wrote the manuscript. CM and SI collected all data regarding the patient's history and clinical course as well as the trends in vital parameters. NP, RM and RF analyzed and interpreted the data regarding the technical aspects of ECMO. All authors read and approved the final manuscript.
